# VapD in *Xylella fastidiosa* Is a Thermostable Protein with Ribonuclease Activity

**DOI:** 10.1371/journal.pone.0145765

**Published:** 2015-12-22

**Authors:** Juliano S. Mendes, André da S. Santiago, Marcelo A. S. Toledo, Luciana K. Rosselli-Murai, Marianna T. P. Favaro, Clelton A. Santos, Maria Augusta C. Horta, Aline Crucello, Lilian L. Beloti, Fabian Romero, Ljubica Tasic, Alessandra A. de Souza, Anete P. de Souza

**Affiliations:** 1 Centro de Biologia Molecular e Engenharia Genética, Universidade Estadual de Campinas, Campinas, SP, Brazil, CEP 13083-875; 2 Departamento de Química Orgânica, Instituto de Química, Universidade Estadual de Campinas, Campinas, SP, Brazil, CEP 13083-970; 3 Centro APTA Citros Sylvio Moreira/IAC, Cordeirópolis, SP, Brazil, 13490-970; 4 Departamento de Biologia Vegetal, Instituto de Biologia, Universidade Estadual de Campinas, SP, Brazil, CEP 13083-862; Centre National de la Recherche Scientifique, Aix-Marseille Université, FRANCE

## Abstract

*Xylella fastidiosa* strain 9a5c is a gram-negative phytopathogen that is the causal agent of citrus variegated chlorosis (CVC), a disease that is responsible for economic losses in Brazilian agriculture. The most well-known mechanism of pathogenicity for this bacterial pathogen is xylem vessel occlusion, which results from bacterial movement and the formation of biofilms. The molecular mechanisms underlying the virulence caused by biofilm formation are unknown. Here, we provide evidence showing that virulence-associated protein D in *X*. *fastidiosa* (Xf-VapD) is a thermostable protein with ribonuclease activity. Moreover, protein expression analyses in two *X*. *fastidiosa* strains, including virulent (Xf9a5c) and nonpathogenic (XfJ1a12) strains, showed that Xf-VapD was expressed during all phases of development in both strains and that increased expression was observed in Xf9a5c during biofilm growth. This study is an important step toward characterizing and improving our understanding of the biological significance of Xf-VapD and its potential functions in the CVC pathosystem.

## Introduction

*Xylella fastidiosa* is a gram-negative, xylem-inhabiting bacterium that is a causal agent in important global diseases in agricultural crops, such as plum, almond, peach, coffee, and grapevine [1]. *X*. *fastidiosa* causes citrus variegated chlorosis disease (CVC) in the orange trees of Brazil, which dramatically reduces fruit production [2,3]. *X*. *fastidiosa* is transmitted by sharpshooter leafhoppers, and once the pathogen enters the xylem, it spreads through channels called bordered pits that connect vessels [4]. The main pathogenic mechanism of *X*. *fastidiosa* occurs when the organism blocks water transport through vessels by forming systemic biofilms. A biofilm is a matrix that is composed of factors including extracellular polymeric substance (EPS), proteins, and nucleic acids [5]. This architecture increases the resistance of the biofilm cells to a wide range of antagonistic agents, such as UV radiation, shifts in pH, osmotic shock, desiccation, antimicrobial compounds, and host defense mechanisms [6]. During the biofilm phase, diffusible signal factors are used in an intercellular communication system called quorum sensing (QS) that, among other functions, controls the expression of virulence factors [7–10]. Biofilm formation occurs through a dynamic and complex mechanism of development in which protein expression can change after only one day of growth [11]. In *X*. *fastidiosa*, biofilm formation on an abiotic surface involves the following five developmental steps: (1) reversible attachment; (2) irreversible attachment; (3) the beginning of maturation, which includes approximately 10 days of growth and the initiation of QS; (4) mature biofilm establishment, which occurs between days 15 and 20; and (5) dispersion, which occurs between days 25 and 30 [12].

Because the Xf9a5c genome has been sequenced [13], many studies have analyzed the open-reading frames (ORFs) in its genome to predict genes that may participate in its pathogenicity and in biofilm formation [14–18]. However, some predicted virulence factors remain unstudied. One predicted virulence factor of interest was found in the plasmid pXF51; in this plasmid, the ORF XFb0051 encodes the 17.6 kDa virulence-associated protein D (Xf-VapD), which contains a predominance of α-helices in its secondary structure [19]. Xf-VapD is predicted to belong to the CRISPR/Cas2 family (Pfam: PF09827). CRISPRs (clustered regularly interspaced short palindromic repeats) are DNA loci that, when associated with Cas proteins, confer resistance to foreign genetic elements, such as plasmids and phages [20]. Cas2 in *Legionella pneumophila*, a gram-negative aquatic bacterium, has been shown to be an important component during infection of amoebal host cells [21]. In addition, several bacterial species also produce the VapD protein. In *Haemophilus influenzae*, the VapXD toxin-antitoxin locus enhanced survival and virulence via a mechanism involving mRNA cleavage during *in vitro* and *in vivo* infection tests [22]. In *Helicobacter pylori*, VapD has been shown to display ribonuclease activity [23], and in *Rhodococcus equi*, it has a β-barrel structure, but its function is unknown [24].

In this study, we performed tests to structurally and functionally characterize Xf-VapD, and we analyzed its expression levels in two strains: 9a5c (virulent) and J1a12 (nonpathogenic). In addition, we demonstrated that Xf-VapD is a thermostable protein with ribonuclease activity. The expression of Xf-VapD was observed in both the Xf9a5c and the XfJ1a12 strain, but a higher level of expression was observed in Xf9a5c during biofilm growth.

## Materials and Methods

### Cloning, expression, and purification

The ORF XFb0051 of *X*. *fastidiosa* (database http://www.xylella.lncc.br/) was amplified using PCR of total DNA (genomic + plasmid) isolated from *X*. *fastidiosa* strain 9a5c, which was used as the template. To modify the original amino acid sequence, we removed the first two residues from its N-terminus and the last 10 residues from its C-terminus. This procedure was necessary because the complete sequence caused instability in crystallographic assays aimed at determining the 3D structure of Xf-VapD (research in progress). Therefore, all experimental procedures were performed using this modified amino acid sequence. The amino acids that were removed did not contain any functional sites. Specific forward (5’- GGTATTGAGGGTCGCGATCGCTGCCTAATCGTTT-3’) and reverse (5’-AGTTAGAGCCTCACGCATTTTCTAGAGAGAACTTCT-3’) primers were designed. The PCR product obtained using these primers was cloned into the pET32Xa/LIC vector (Novagen; USA) and used to transform the *E*. *coli* strains DH5-α and BL21 (DE3) (Novagen; USA) to induce heterologous protein expression. Xf-VapD protein expression and purification were performed according to the methods described in [19] and by using an FPLC ÄKTApurifier GE^®^ for the chromatography steps. The purified protein was analyzed using 12.5% SDS-PAGE [25].

### CD spectroscopy and CD thermal denaturation

The purified Xf-VapD protein (2.0 μM) was dialyzed against 20 mM Tris-HCl and 10 mM NaCl, pH 7.5, and evaluated using a JASCO J-810 circular dichroism (CD) spectropolarimeter (Japan Spectroscopic; Tokyo, Japan) that was coupled to a temperature controller. The sample was contained in a 0.1 cm quartz cuvette. The CD spectra were recorded in a measurement range of 200 nm–260 nm at 25°C. The results were analyzed using the DichroWeb server [26]. For the denaturation assays, Xf-VapD CD was measured at intervals from 20°C–110°C. After a final measurement at 110°C, the sample was cooled back down to 20°C. The sample was then contained in a 1 cm quartz cuvette.

### RNase activity assay

Xf-VapD recombinant purified protein (1.0 mg/mL) that was suspended in a buffer containing 50 mM Tris and 300 mM NaCl at pH 7.5 was incubated with a modified RNA oligonucleotide that emits a green fluorescence when cleaved by RNase (RNaseAlert^®^ QC System) at 37°C for 180 minutes. In this assay, we used two temperatures, 37°C and 90°C, for the Xf-VapD activity studies. The first temperature was the same as the temperature that was used for the recombinant expression of Xf-VapD, and the second temperature was used to test the enzyme’s activity at a high temperature. For the 90°C tests, the protein was denatured for 30 minutes at 90°C and then centrifuged (16,110 x g, 25°C, 3 minutes). The supernatant fraction was incubated at 37°C with the modified RNA oligonucleotide for 180 minutes. A positive control (RNase A) and negative controls (Xf-VapD purification buffer and nuclease-free water) were used in the assay. The measurements were performed in duplicate for each experimental sample using a fluorometer with excitation and emission wavelengths of 490 nm and 520 nm, respectively.

In addition, Xf-VapD RNase activity was visualized using 1% agarose gels. For these experiments, total RNA was obtained from the E. coli strain BL21(DE3) using TRI Reagent^®^ solution (Thermo Fisher Scientific, USA) and purified using an RNeasy^®^ Mini Kit (Qiagen, GER). Aliquots containing 1.0 μg of total RNA were incubated for 1 h at 25°C with 1 μM, 5 μM, 10 μM and 20 μM of Xf-VapD. In addition, a DNase activity test was performed. For these experiments, long and a short double-stranded DNA (dsDNA) and single-stranded DNA (ssDNA) were used. A long dsDNA (2 kb) template corresponding to multicopper oxidase was obtained using metagenomic prospection in Caatinga soil and prepared using PCR with specific primer pairs (Fwd, [5’-ACTGCATATGTTCGTTCAGCC-3’] and Rev, [5’-TTCGAGCTCGAGGAGCTACC-3’]). A short dsDNA (20 bp) [5’-AGGCAGGACTATGGAGGCGA-3’] and a ssDNA (26 nt) [5’-CCACATATGACAAACCTATTACAAGT-3’] were commercially synthesized. Aliquots containing 2 μg of dsDNA or ssDNA were incubated for 1 h at 25°C with 20 μM or 50 μM of Xf-VapD. The dsDNA (2 kb) was visualized using a 1% agarose gel, while the dsDNA (20 bp) and the ssDNA (26 nt) were visualized using 20% acrylamide gels.

### Expression profiles of Xf-VapD in different phases of biofilm formation

The expression level of Xf-VapD in biofilm and planktonic *X*. *fastidiosa* cells was determined using western blot analysis with a specific anti-rabbit IgG polyclonal antibody against Xf-VapD that was synthesized by Rhea Biotech, Brazil. Xf9a5c and XfJ1a12 cells were grown in periwinkle wilt (PW) medium [27] for seven days until they reached *A*_600_ = 0.7. A total volume of 2 mL of each suspension was then inoculated into a 125-mL flask containing 50 mL of PW medium. The flasks were maintained at 28°C with rotary agitation (120 rpm) for three, five, 10, 20, 30 days. An analysis of the XfJ1a12 biofilm cells was not performed because this strain forms a very thin biofilm *in vitro*. The extraction of total protein from Xf9a5c cells (biofilm and planktonic) and XfJ1a12 cells (only planktonic) was performed according to the protocol described in [12]. The total protein was quantified using the Bradford method and normalized to equal concentrations. A total of 5.0 μg of protein was separated using 12.5% SDS-PAGE. The acrylamide gel was then transferred to a nitrocellulose membrane using a Semi-Dry Transfer Cell (BioRad, CA, USA). The membrane was blocked in a 1% casein solution and incubated first with a specific antibody against Xf-VapD (dilution 1:1,000) and then with a secondary antibody, anti-rabbit IgG conjugated to alkaline phosphatase enzyme (dilution 1:8,000). BCIP/NBT Color Development Substrate (Promega) was used to detect alkaline phosphatase activity and visualize the bands. The bands were quantified using ImageJ software, and the statistical significance of the results was analyzed using two-tailed Student’s *t*-tests (*p*<0.05 was defined as indicating statistical significance). The experiments were performed in triplicate.

### Alignment, phylogenetic analysis, and protein model prediction

The VapD amino acid sequences were used to search the NCBI data bank. To analyze their alignment, the sequences were separated according to their percentage of identity to Xf-VapD (accession number Uniprot/NCBI: Q9PHF6/AE003851_51). Group 1 shared identity with Xf-VapD was above 50%, group 2 was from 49 to 25%, and group 3 was below 25%. VapD sequences were obtained for the following species: *X*. *fastidiosa* strain Ann-1 (A0A060H4 L5/AIC11724.1), *Gallibacterium anatis* (A0A0A2YXK7/WP_018347295), *Aggregatibacter actinomycetemcomitans* (G4A5B7/ EGY35307), *Neisseria meningitides* (L5UTE0/WP_002251489); *Escherichia coli* (C6CFU7/WP_001402204), *Xanthomonas campestris* (G2 LRG4/ WP_039405661), *Haemophilus influenzae* (A4NV08/WP_013526240), *Helicobacter pylori* (A0A0B5FMI2/ 3UI3_B), and *Rhodococcus equi* (B4F3C5/4CSB). For the phylogenetic analysis, the VapD sequences were aligned using TranslatorX software [28] with the Muscle Algorithm. All sequences were translated into amino acids, which were then aligned and further back-translated into nucleotide sequences. Both the amino acid and nucleotide alignments were used to infer phylogenetic relationships between all of the identified VapD proteins. A neighbor-joining phylogenetic tree was constructed using MEGA 5.2 [29] and inferred using uncorrected p-distances and the partial deletion of sites (50%). Branch supports were accessed through 1,000 bootstrap replicates. Bayesian inference analyses were performed using MrBayes v3.1.2 [30]. The modeling of nucleotides (+I+G) and amino acids (GTR+G) was performed using MrAIC [31] and ProtTest [32], respectively. *Rhodococcus equi* was used as the outgroup for the phylogenetic tree. The final protein model was obtained using the protein homology/analogy recognition engine server Phyre V 2.0 and the I-TASSER server [33,34].

### Ethics statement

We confirm that no specific permits were required for the described field studies. Sample collection was carried out at two research institutions (the University of Campinas-UNICAMP and the Citriculture Center in Cordeirópolis, São Paulo, Brazil). The bacteria were grown in a laboratory that was certified biosafety level 1. In addition, we confirm that this manuscript is the result of a basic research project that was developed mainly at the university and that was funded primarily by public funding agencies with the aim of developing new knowledge. The generated results should therefore be shared with the scientific and technological community through Open University theses and manuscripts that are published in conventional scientific journals. In this sense, we would like to state that we fully adhere to all of the PLOS ONE policies regarding the sharing of data and materials. We confirm that this study did not involve endangered or protected species.

## Results

### Alignment and phylogenetic analysis

**Fig 1:**
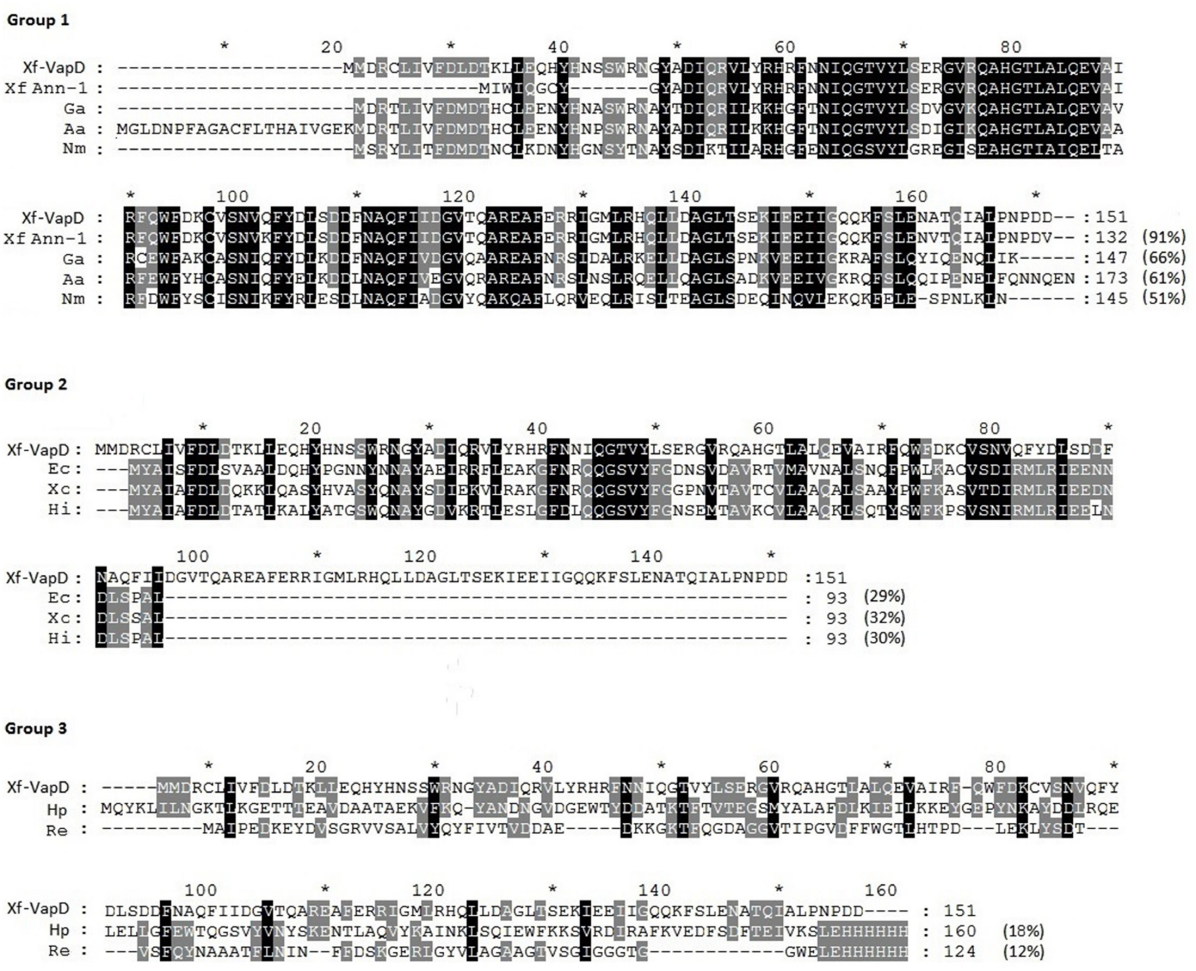
Multiple sequence alignments to VapD proteins. Group 1: The *X*. *fastidiosa* strains 9a5c and Ann-1 (Xf9a5c and XfAnn-1, respectively), *G*. *anatis* (Ga), *A*. *actinomycetemcomitans* (Aa), and *N*. *meningitides* (Nm). Group 2: *E*. *coli* (Ec), *X*. *campestris* (Xc), and *H*. *influenza* (Hi). Group 3: *H*. *pylori* (Hp) and *R*. *equi* (Re). The identity values for Xf-VapD are shown in parentheses at the end of each sequence.

**Fig 2:**
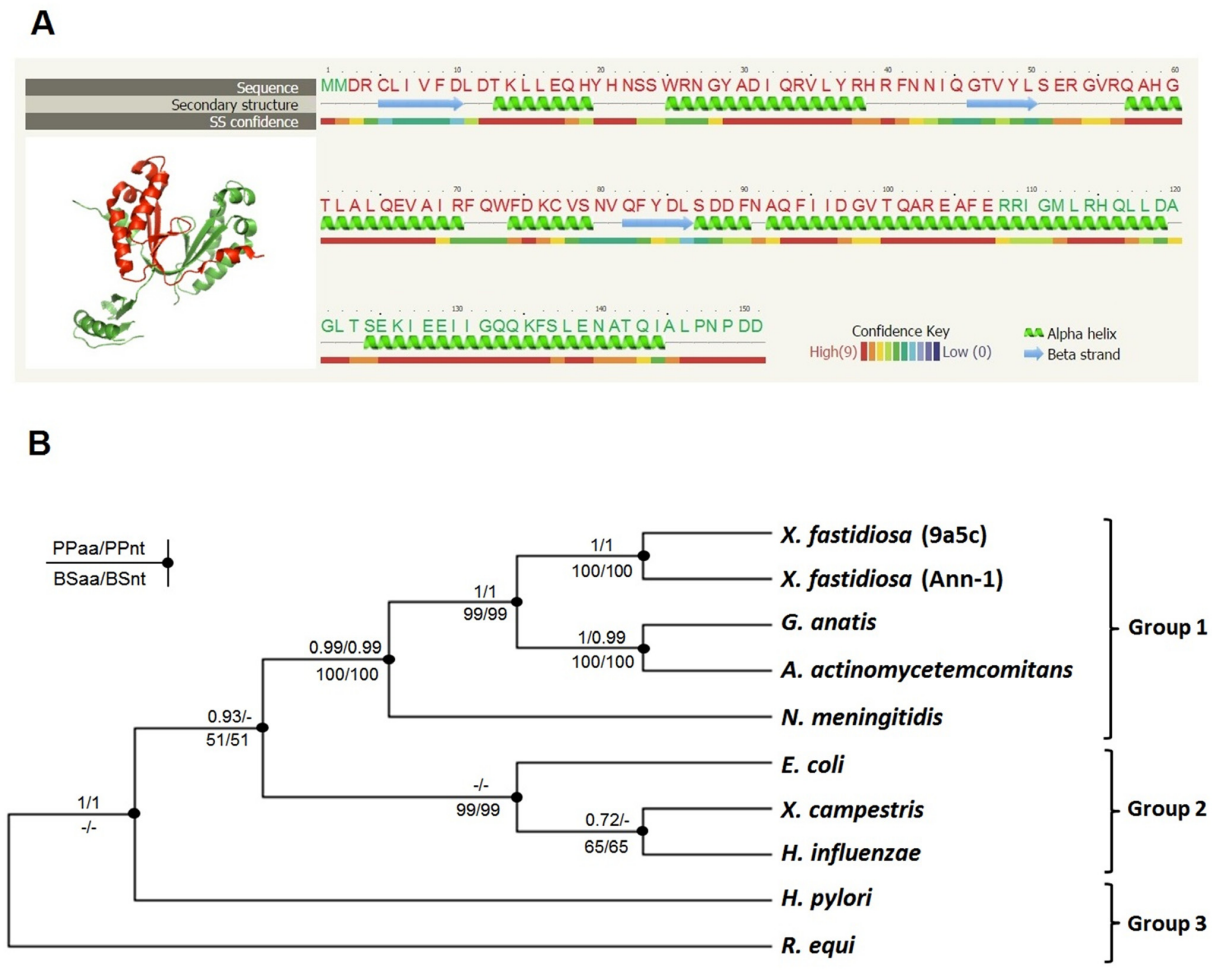
Structural and phylogenetic analysis of Xf-VapD. (A) The prediction of the 3D structure of Xf-VapD was performed using Phyre V 2.0 (red cartoon). A model was obtained with 66% of the Xf-VapD sequence and 100% confidence using the single highest scoring template. The structure was edited using PyMol. On the right, the amino acid sequence is shown with the respective secondary structure and confidence. The amino acid residues highlighted in red correspond to the area in the red cartoon shown in the 3D structure. (B) The neighbor-joining consensus tree inferred for the amino acid sequences of VapD. The values above the branches indicate the Bayesian posterior probabilities for the amino acids (PPaa) and nucleotides (PPnt). The values below the branches indicate the results of the neighbor-joining method for the amino acids (BSaa) and nucleotides (BSnt) following 1,000 bootstrap replicates. The minus symbol (‒) indicates that no support was reached for this node. The groups are the same as those in Fig 1.

The Xf-VapD amino acid sequence showed high identity to the VapD sequences of several different bacterial species (Fig 1). The VapD sequences analyzed in groups 1, 2 and 3 (except those of *R*. *equi*) were found to be related to the Cas2 family of CRISPR/Cas system-associated proteins. Using the PSIPRED secondary structure prediction method v3.3 [35], we found that the amino acids have a secondary structure that is mainly composed of α-helices with a small proportion of β-sheets. In group 3, the VapD sequences from *H*. *pylori* and *R*. *equi* exhibited low identity with Xf-VapD (18% and 12%, respectively), but their recently published structures revealed that they display interesting characteristics. VapD in *R*. *equi* has β-barrel structural features (PDB ID. 4CSB), whereas VapD in *H*. *pylori* has α-helix and β-sheet structural features and endoribonuclease activity (PDB ID 3UI3) [23,24]. In a preliminary investigation of the structure of Xf-VapD, we used the protein homology/analogy recognition engine Phyre V 2.0 [33] to identify the closed structure of Xf-VapD. We found that 66% of its sequence (Position: 5–107 or CLI—AFE) had 100% confidence (the probability that the sequences of Xf-VapD and the template are homologous) to VapD in *H*. *pylori* (Fig 2A). This structural prediction was confirmed using the I-TASSER server [34]. Phylogenetic analyses showed that the Xf-VapD and VapD in groups 1 and 2 and *H*. *pylori* are homologues and form a distinct clade, while the Xf-VapD and VapD of *R*. *equi* do not appear to share the same evolutionary course (Fig 2B).

### Recombinant Xf-VapD expression and purification

**Fig 3:**
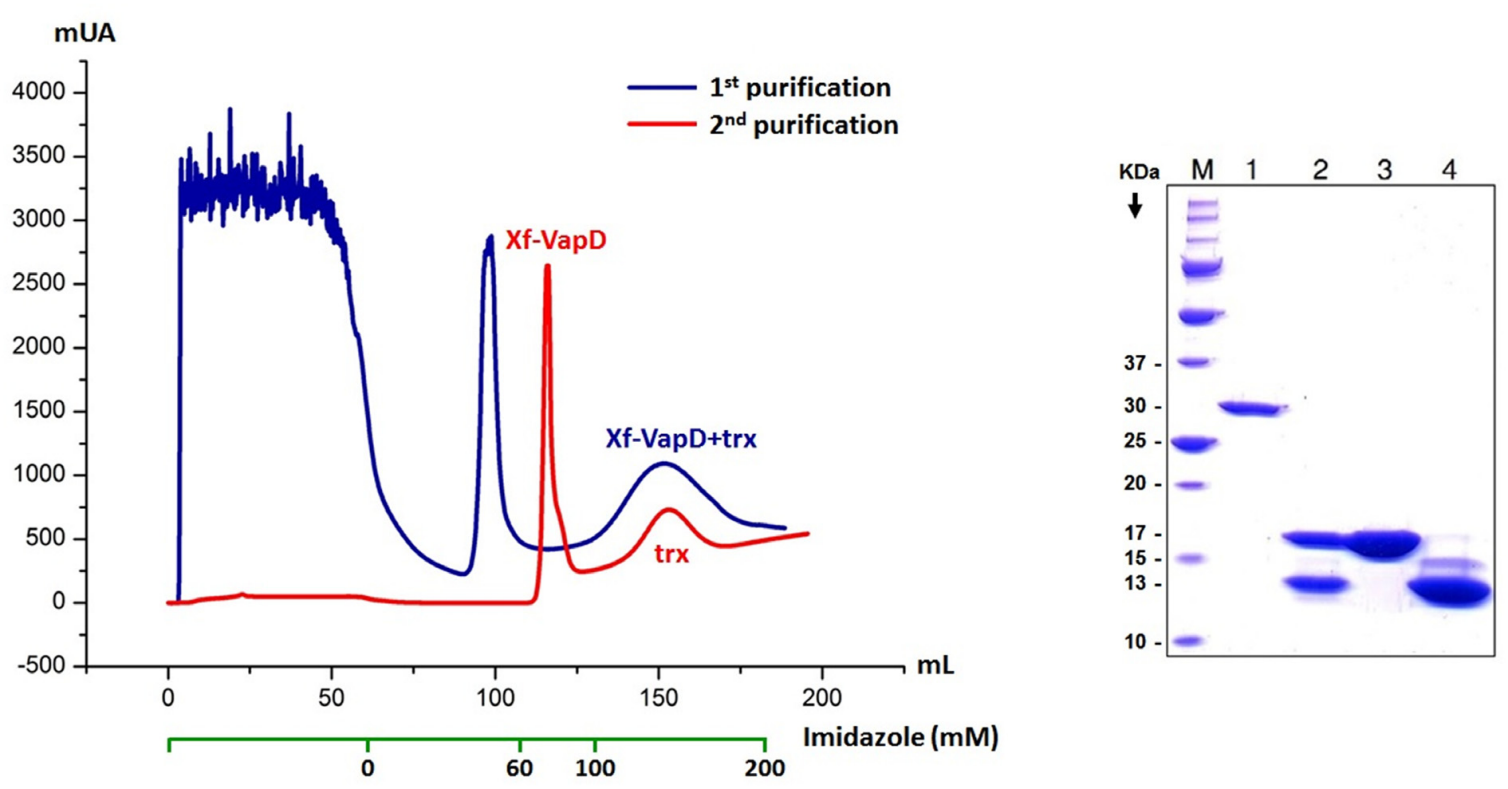
Purification of recombinant Xf-VapD. A chromatogram showing the two steps used in the purification of Xf-VapD: Xf-VapD fused with a trx-tag was obtained first, and then trypsin cleavage was used to remove the trx-tag. On the right, a 12.5% SDS-PAGE is shown for the Xf-VapD purification process. The lanes are as follows: M, protein marker; 1, Xf-VapD fused to trx-tag (31 kDa); 2, Xf-VapD and trx-tag after cleavage with trypsin; 3, totally purified Xf-VapD (16.5 kDa); and 4, trx-tag (~13 kDa). The gel was stained using Coomassie brilliant blue.

Xf-VapD was successfully expressed and purified using the well-established protocol that is described in [19]. The complete amino acid sequence was modified by removing amino acids to perform the posterior crystallographic assay, as described in the experimental procedures. In the first step of the purification, Xf-VapD fused to a thioredoxin (trx-tag) was obtained with a molecular mass of approximately 30 kDa. In the second step, after cleavage with a 1 mg/mL trypsin solution, purified Xf-VapD (6.85 mg/L of bacterial culture) was obtained with a molecular mass of 16.5 kDa (Fig 3). Protein identity was confirmed using mass spectrometry.

### Secondary structure and thermo stability assay of Xf-VapD

**Fig 4:**
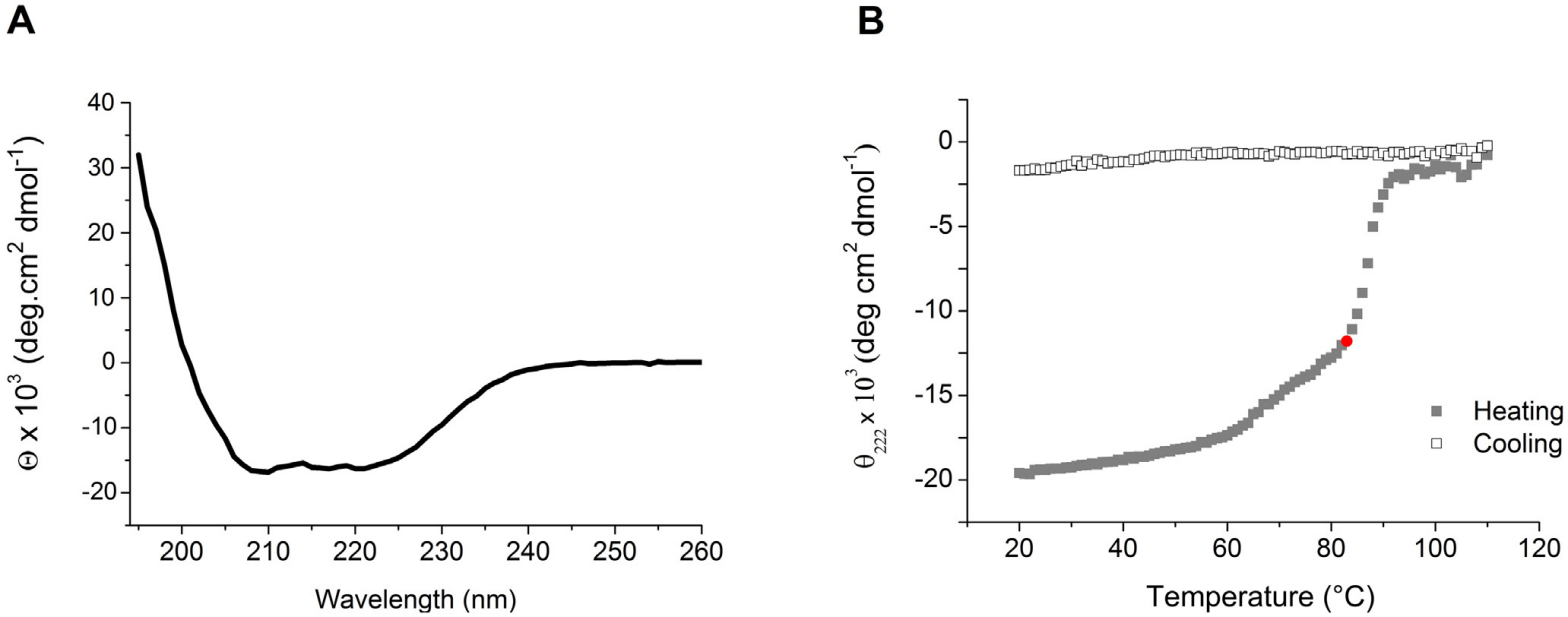
Secondary structure and thermo stability assay of Xf-VapD. (A) Circular dichroism spectrum (200 nm–260 nm) of the recombinant purified Xf-VapD. The secondary structure of the protein was found to be 69% α-helices, 19% β-sheets, and 12% random coils. (B) Thermal unfolding/refolding of Xf-VapD. At 83°C during the heating phase, 50% of the proteins were unfolded (red marker). The conformational structure of the protein was not restored during the cooling phase.

The secondary structure of Xf-VapD was determined using CD spectroscopy data. This analysis revealed a high proportion of α-helices (69%) and a minor β-sheet component (19%) (Fig 4A). CD spectroscopy was also used to determine the thermodynamic stability of Xf-VapD. For these experiments, the recombinant protein was tested along a temperature gradient ranging from 20°C to 100°C. During the heating phase, Xf-VapD had shown a capacity to withstand a high temperature and a T_m_ of 83°C (Fig 4B). The cooling phase did not result in the refolding of Xf-VapD. This irreversible denaturation generally occurs as a result of the aggregation of unfolded polypeptides [36].

### Functional characterization of Xf-VapD

**Fig 5:**
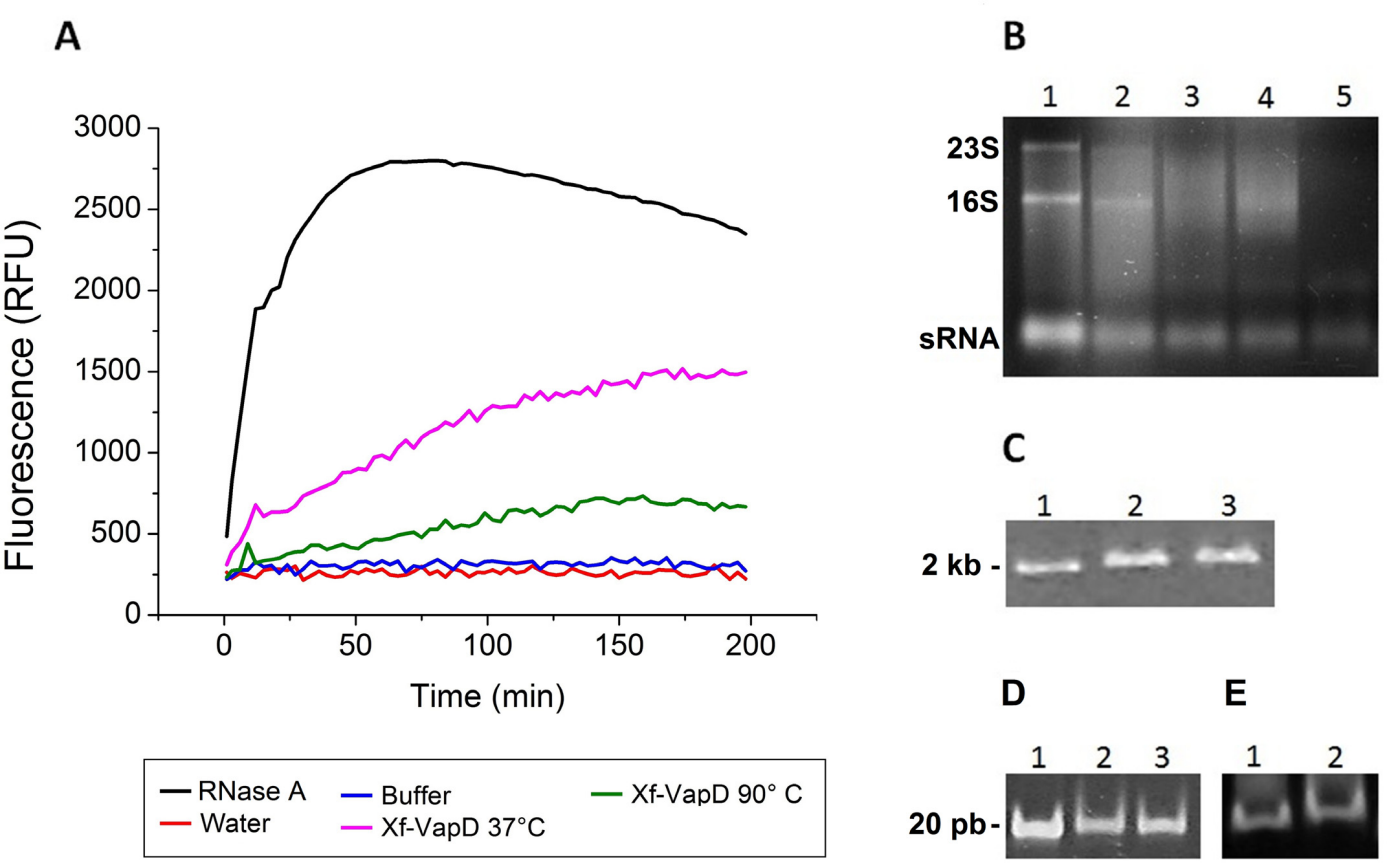
Enzymatic activity assay of Xf-VapD. (A) The lanes contain one positive control, two negative controls, and the Xf-VapD samples that were treated at 37°C and 90°C. The values are shown in RFU/time units. The curves show that Xf-VapD demonstrated ribonuclease activity at both 37°C, and after heating to 90°C. (B) Lane 1 contains RNA alone, and lanes 2 to 5 contain RNA with Xf-VapD (1 μM, 5 μM, 10 μM and 20 μM, respectively). (C) Lane 1 contains dsDNA (2 kb) alone, and lanes 2 to 3 contain dsDNA (2 kb) with Xf-VapD (20 μM and 50 μM, respectively). (D) Lane 1 contains dsDNA (20 bp) alone, and lanes 2 to 3 contain dsDNA (20 bp) with Xf-VapD (20 μM and 50 μM, respectively). (E) Lane 1 contains ssDNA (26 nt) alone, and lane 2 contains ssDNA (26 nt) with Xf-VapD (50 μM).

Ribonuclease activity has been observed in VapD and CRISPR-associated Cas2 proteins [21,23]. Thus, a ribonuclease activity assay was performed using an RNase detection kit (RNaseAlert^®^ QC System, Life Technologies, USA) to determine whether Xf-VapD has a similar protein function. This kit is composed of a modified RNA that emits green fluorescence when cleaved by RNase. Activity was tested at two temperatures (37°C and 90°C). Xf-VapD showed ribonuclease activity at both temperatures. Heating the test solution to 90°C resulted in a 50% reduction in the maximum relative fluorescence units (RFU) compared to the RFU observed at 37°C (Fig 5A), which supported the results of the CD unfolding experiments. The positive control (ribonuclease A) and the negative controls (Xf-VapD buffer and nuclease-free water) corresponded to the results observed in the quality assay. The basal values in the negative controls (close to 250 RFU) were normal according to the kit protocol. *In vitro* tests performed using total RNA obtained from *E*. *coli* also demonstrated the ribonuclease activity of Xf-VapD at different protein concentrations (Fig 5B). In addition, interaction tests using long (2 kb) and short (20 bp) dsDNA and ssDNA (26 nt) were performed. However, Xf-VapD did not show a level of activity that is equivalent to the level found using RNA substrates (Fig 5C–5E).

### Analysis of Xf-VapD expression in different biofilm and planktonic growth phases

The function of VapD remains unknown in many organisms. As shown in Fig 5, Xf-VapD demonstrated ribonuclease activity, which is an important function that has been observed in members of the CRISPR/Cas2 system [37]. *X*. *fastidiosa* has two modes of growth. Planktonic growth occurs when the cells are dispersed in the media, while biofilm growth involves the cells adhering to biotic or abiotic surfaces and multiplying to form microcolonies. Biofilm growth is an important feature of virulence symptoms in CVC [9]. Thus, we next evaluated protein expression levels in the Xf9a5c and XfJ1a12 strains during the following five growth timepoints: reversible attachment (at three days), irreversible attachment (at five days), initial maturation (at 10 days), mature biofilm (at 20 days), and dispersion (at 30 days). Planktonic cells were also analyzed at the same time points. An important feature of the nonpathogenic XfJ1a12 strain is its inability to form a thick biofilm to induce CVC symptoms when inoculated in citrus plants [38,39]. Thus, we used this nonpathogenic strain as a control to determine whether there is a difference in Xf-VapD expression between virulent and non-virulent strains because Xf-VapD has been associated with virulent characteristics.

**Fig 6:**
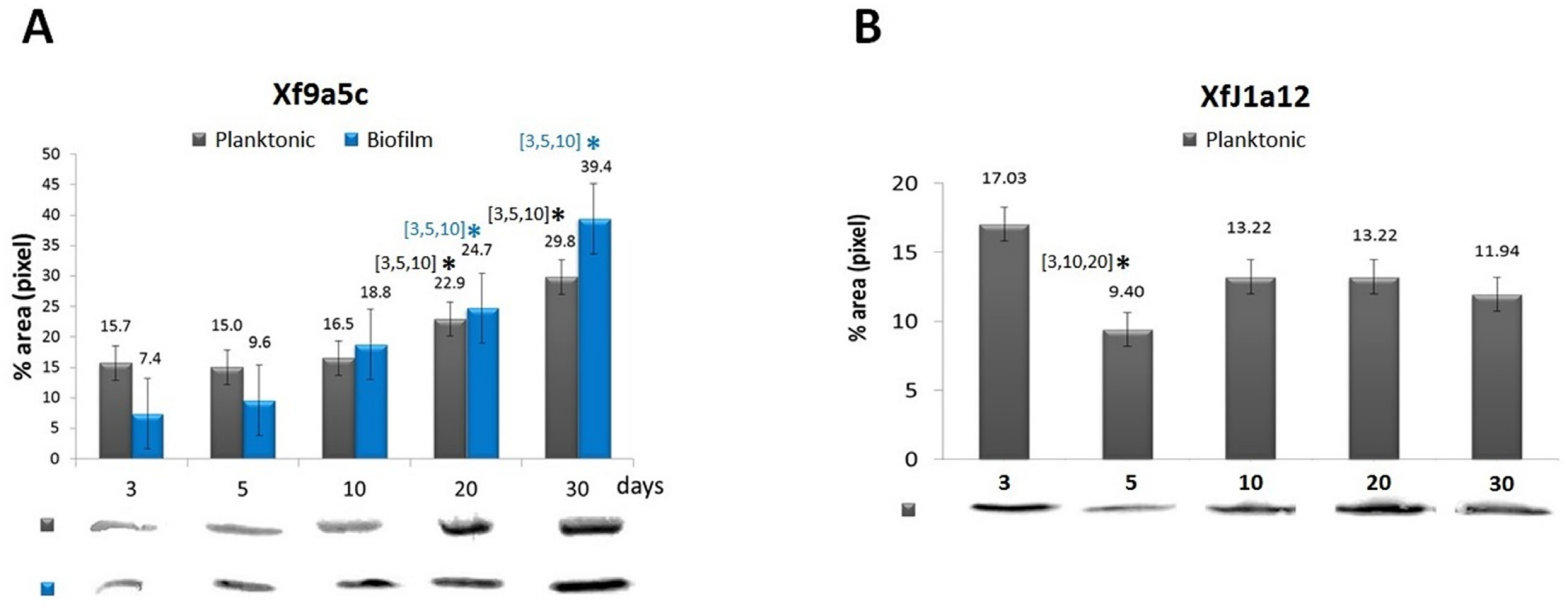
Western blot analyses of Xf-VapD expression in *X*. *fastidiosa* cells. (A) Strain 9a5c in planktonic and biofilm growth. (B) Strain J1a12 in planktonic growth. The band values were obtained using ImageJ software. The values above the bars indicate the average of three biological replicates. The error bars indicate the standard errors of the means. The asterisk (*) indicates a significant difference compared to the days of growth indicated in the brackets (Student’s *t*-test, *p*<0.05).

During the biofilm growth induced by Xf9a5c, an increase in Xf-VapD expression was detected, and this increase was correlated with the development of the biofilm (Fig 6A). The expression of Xf-VapD was significantly increased in this strain between days 20 and 30, which are timepoints that correspond to the mature biofilm and dispersion stages, respectively. In these cells, during the planktonic growth phase, no significant difference in Xf-VapD expression was observed between days three, five and 10, but Xf-VapD expression was increased on days 20 and 30 of growth. However, in the XfJ1a12 cells, while Xf-VapD was expressed during all stages of planktonic growth, no significant difference was found between its expression on days 20 and 30 of growth, unlike what was observed in the virulent strain (Fig 6B). A decrease in VapD expression was observed in this strain on only the 5^th^ day.

## Discussion

The main mechanism of the pathogenicity of *X*. *fastidiosa* lies in its capacity to form biofilms, which cause vascular occlusions inside xylem vessels, leading to water stress [40]. However, many of the molecular mechanisms involved in its virulence have not been fully explored. Interestingly, 64 ORFs have been predicted in the pXF51 plasmid of Xf9a5c. Among these, only one, Xfb0051, which encodes Xf-VapD, has been associated with the pathogenesis caused by vascular occlusions [13,41]. Xf-VapD is characterized as a virulence-associated protein, but its contributions to virulence and pathogenicity are not understood. However, by definition, virulence-associated genes must act in one of three ways: by regulating the expression of virulence genes, by activating virulence factors via translational modification, processing or secretion, or by co-activating true virulence factors [42].

In the NCBI data bank, Xf-VapD is annotated as the CRISPR system-associated protein Cas2, which has been associated with endoribonuclease activity. In addition to conferring immunity against phages and plasmids, the Cas2 proteins are virulence factors in *L*. *pneumophila* [21]. Most VapD proteins in bacterial species have unknown functions. The PDB data bank (http://www.rcsb.org) contains only two resolved VapD structures, 3UI3 and 4CSB [23,24]. However, these two structures are very different, suggesting that they have distinct functions. Xf-VapD has high identity with VapD in several bacteria. Phylogenetic analyses have shown that the VapD sequences in *H*. *pylori* and *R*. *equi* are not closely related with Xf-VapD (Fig 2B). However, an *in silico* prediction showed that Xf-VapD has high structural and functional homology with VapD in *H*. *pylori* (3UI3) (Fig 2A). The structural and functional similarities between Xf-VapD and VapD in *H*. *pylori*, in addition to the high identity observed in these sequences in other species, suggest that a change occurred along the evolutionary course of VapD that led to the preservation of functions between homologous species.

In the present study, we demonstrate that Xf-VapD is a thermostable protein (Fig 4B). Several factors are associated with thermal stability, including hydrophobic interactions, ionic interactions, and amino acid preference. However, the specific contributions of these factors vary among different proteins, and this makes them difficult to explore [43,44]. The necessity of any one of these characteristics in a protein is usually associated with the maintenance of structural folds and functional activities under extreme environmental conditions [45]. The hostile environment inside xylem vessels is caused by factors including a low concentration of solutes in the sap and oscillations in these concentrations [46], and these may require a pathogen to be more resistant to antagonistic agents. In this way, protein stabilization by hydrophobic interactions can, for example, play an important role in the spread of bacteria within a host.

Heat resistance is a common characteristic of ribonucleases [47–49]. This motivated us to investigate this heat resistance in Xf-VapD. Enzymatic tests showed that at 37°C, Xf-VapD has ribonuclease activity. After heating the solution to 90°C, Xf-VapD maintained 50% of its activity. Ribonucleases are associated with the establishment of virulence in many pathogenic bacteria, in which they act to regulate non-coding RNAs at several levels, including their biogenesis, processing and turnover [50,51]. In *Vibrio cholera*, for example, the YbeY protein is a ribonuclease that is essential for virulence and stress regulation [52], whereas ribonuclease R is a virulence factor that is important during the first steps of infection, adhesion and invasion of eukaryotic cells by *Campylobacter jejuni* [53].

Our preliminary investigation of the structural and functional characteristics of Xf-VapD is an important step towards understanding the role of this protein. In this study, we show that the secondary structure of Xf-VapD is 69% α-helices and 19% β-sheets. These results support previous structural predictions in which PSIPRED was used. Moreover, a new strategy for expressing a modified amino acid sequence of the protein was necessary to avoid instabilities that were observed during crystallographic assays (data not shown). Research is in progress to determine the 3D structure of Xf-VapD.

The expression of Xf-VapD was observed in Xf9a5c and XfJ1a12 cells. In prokaryotes, ribonucleases participate in the transcriptional and post-transcriptional control of gene expression, in regulating plasmid replication, and in maintaining the transfer of genetic material by controlling the levels of antisense RNAs and small RNAs [54–57].

Interestingly, differences in the expression profile of Xf-VapD between virulent and nonpathogenic strains were observed in planktonic cells. While Xf-VapD expression in the virulent strain, Xf9a5c, increased as cell growth increased (Fig 6A), the non-virulent XfJ1a12 cells did not exhibit similar behavior (Fig 6B). An analysis of Xf-VapD levels in the biofilms of XfJ1a12 cells was not performed because this strain forms a very thin biofilm *in vitro*. During Xf9a5c biofilm growth, an increase in the expression of Xf-VapD was observed from day 20 to day 30 during both biofilm and planktonic growth (Fig 6A). These results support the findings of studies of VapD gene expression in *X*. *fastidiosa* [58].

The results of this study indicate that there is a need to increase the number of studies investigating Xf-VapD and the mechanisms that contribute to its virulence. These should include studies that use site-directed mutagenesis assays. The functional characterization of Xf-VapD as a thermostable ribonuclease that is primarily expressed in the mature biofilms of the virulent 9a5c strain is an important step toward increasing our understanding of the biological significance of Xf-VapD in this pathosystem.
